# Enhancing Mechanical Properties of Q&P Steel Through Tailoring Film-like Retained Austenite Morphology via Heating Rate Optimization

**DOI:** 10.3390/ma18204815

**Published:** 2025-10-21

**Authors:** Shengwei Wang, Mingyue Yang, Mengxiao Chen, Yuhe Huang, Shuize Wang, Xinping Mao

**Affiliations:** 1 Institute for Carbon Neutrality, University of Science and Technology Beijing, Beijing 100083, China; 2 Shanghai Key Laboratory of Materials Laser Processing and Modification, School of Materials Science and Engineering, Shanghai Jiao Tong University, Shanghai 200240, China; 3 Research Institute, Baoshan Iron and Steel Co., Ltd., Shanghai 201999, China; 4 Institute of Steel Sustainable Technology, Liaoning Academy of Materials, Shenyang 110000, China

**Keywords:** Q&P steel, heating rate, mechanical properties, microstructure, film-like retained austenite

## Abstract

In this study, by regulating the heating rate, we have optimized the morphology of retained austenite in a quenching and partitioning (Q&P) steel with initial microstructure consisting of lath martensite. By optimizing the heating rate to 1 °C/s during the heating stage from 700 °C to 830 °C, the formation of blocky austenite and the coarsening of film-like austenite during the two-phase region annealing process were prohibited. Ultimately, this resulted in the production of Q&P steel containing a significant volume fraction (19.0%) of fine film-like retained austenite. This fine and film-like retained austenite exhibits higher stability than blocky austenite, exhibiting an active transformation-induced plasticity (TRIP) effect over a broad strain range. This results in excellent mechanical properties characterized by a high product of tensile strength and elongation (34.1 GPa·%).

## 1. Introduction

To comply with stringent requirements for lightweight design and lower emissions, the automotive industry continuously seeks advanced high-strength steels (AHSSs) offering higher performance [1,2,3]. Quenching and partitioning (Q&P) steels, a notable third-generation AHSS, are highly regarded for their excellent mechanical property balance and production efficiency [4,5,6,7,8]. A critical contributor to this performance is the transformation-induced plasticity (TRIP) effect in retained austenite (RA) during deformation [1,3,9,10]. By promoting a high and sustainable strain-hardening capability, the TRIP effect delays necking to higher strains, enabling simultaneous gains in uniform elongation and tensile strength [11,12,13,14,15]. The morphology of RA dictates its stability; the film-like morphology is notably more stable than the blocky one, which transforms quickly under minor deformation and thus minimally contributes to the TRIP effect [16,17,18]. However, achieving a high-volume fraction of film-like RA in Q&P steels is still challenging.

This difficulty arises because, prior to quenching, most austenite grains in the two-phase region exhibit a blocky morphology due to the austenitization characteristics of the ferrite–pearlite microstructure [19], which is commonly used as the initial microstructure for Q&P steel [20,21,22]. Upon cooling to the quenching temperature, the larger blocky austenite grains, due to their lower stability [23], undergo complete or partial transformation into martensite and a limited fraction of film-like austenite grains are retained between the martensitic laths. Conversely, smaller blocky austenite grains, being more stable [23], resist martensitic transformation. These untransformed austenite grains, predominantly blocky in shape, absorb carbon atoms from the martensite during the partitioning process [4], becoming more stable and ultimately are retained at room temperature. Therefore, how to obtain more film-like RA through new mechanisms and approaches is a key issue to consider.

Efforts have been made to overcome this issue. Chao et al. [24] utilized rapid heating of Mn-partitioned lamellar pearlite to achieve a chemically patterned austenite which, upon quenching, yielded a microstructure dominated by film-like RA. By tailoring the martensitic pre-microstructure via annealing, Sugimoto et al. [14,25,26] proved that film-like RA can be obtained in TRIP steels, due to the nucleation and growth of film-like austenite between the martensitic laths during the annealing process. Inspired by these findings, martensitic pre-microstructures have been introduced into the study of Q&P steel to obtain film-like RA [16,27,28,29]. However, a non-neglectable issue is the unavoidable formation of large-size blocky RA while engineering the film-like RA into the final microstructure. Ding et al. [30] mitigated this by leveraging pre-existing interlath-retained austenite as preferential nucleation sites, which suppressed blocky austenite formation through phase equilibrium considerations. While the existing methods to obtain a high-volume fraction of film-like RA either require adding fast heating equipment to the current production line or involve complex heat treatment processes, the wide-ranging application of high-performance Q&P steel with film-like RA still remains challenging.

In this work, we propose a straightforward and efficient strategy to produce martensitic pre-microstructure Q&P steel using the heating capacity of existing industrial continuous annealing production lines, eliminating the need for fast heating equipment. By reducing the heating rate to 1 °C/s, we delay the onset of the high-temperature range that is conducive to the rapid growth of blocky austenite. This provides sufficient time for the slow-growing film-like austenite, which has numerous nucleation sites between the martensitic laths, to grow in the low-temperature region. Consequently, the formation of blocky austenite is inhibited, ultimately leading to the generation of a significant quantity of film-like RA in the final microstructure. This distinctive microstructure imparts an excellent mechanical property synergy, characterized by a superior balance between high strength and considerable ductility.

## 2. Materials and Methods

**Figure 1:**
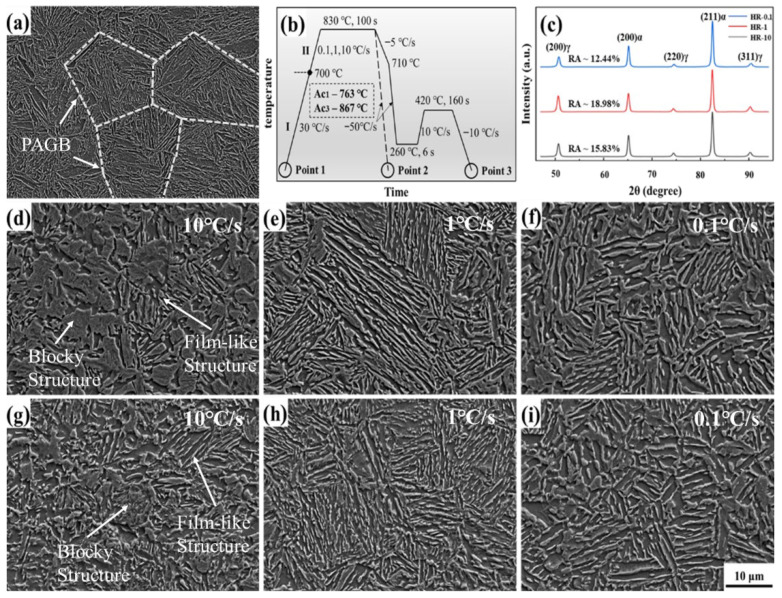
SEM micrographs of the microstructure and schematic representation of the Q&P heat treatment process. (**a**) Initial lath martensitic structure corresponding to Point 1 in (**b**); (**b**) schematic of the quenching and partitioning cycle; (**c**) XRD patterns and retained austenite (RA) volume fractions for different samples; (**d**–**f**) microstructures at Point 2; and (**g**–**i**) at Point 3 in (**b**), obtained under heating rates of 10 °C/s, 1 °C/s and 0.1 °C/s, respectively.

The material investigated in this study was a Fe-0.2C-1.75Si-2.0Mn (wt.%) steel, supplied as a 1.6 mm thick hot-rolled plate by Baowu Group (Shanghai, China) via their thin slab casting and rolling (TSCR) route. Dilatometric analysis was performed using a DIL805A instrument (BAHR-Thermoanalyse GmbH, Hüllhorst, Germany) to determine the Ac1 and Ac3 temperatures to be 763 °C and 867 °C, respectively. To establish a uniform initial condition, the plates were austenitized at 900 °C for 5 min followed by fast cooling (50 °C/s) to room temperature, resulting in a fully martensitic starting microstructure. The quenching and partitioning (Q&P) heat treatment cycles, schematically illustrated in Figure 1b, were implemented on a continuous annealing simulator (CCT-AY-II, Ulvac-Riko INC., Yokohama, Japan). All specimens were initially heated to 700 °C at 30 °C/s. The critical second stage involved heating to the intercritical annealing temperature of 830 °C at different rates (0.1, 1, or 10 °C/s), followed by a 100 s hold to develop a ferrite–austenite duplex structure. Subsequent steps included cooling to 710 °C at 5 °C/s; quenching to 260 °C at 50 °C/s with a 6 s hold to form a controlled amount of primary martensite; and a partitioning step at 420 °C for 160 s (heating rate: 10 °C/s) to enrich the austenite with carbon. Finally, the samples were cooled to room temperature at 10 °C/s. The samples are designated as HR-10, HR-1 and HR-0.1 based on their respective heating rates in stage II. The key temperatures for the Q&P process were selected to align with the standard industrial practice of our partner company, Baowu Group (Shanghai, China), to ensure the relevance and comparability of the results. The austenitization temperature of 830 °C was applied to achieve a target dual-phase microstructure of ferrite and austenite. The quenching temperature of 260 °C was chosen to obtain a controlled fraction of fresh martensite, and the partitioning temperature of 420 °C was selected to enable carbon partitioning while suppressing carbide formation. Microstructural characterization was performed using a Zeiss Gemini 500 field-emission scanning electron microscope (SEM) (Carl Zeiss AG, Jena, Germany). Electron backscatter diffraction (EBSD) analysis was conducted on the same instrument equipped with an Oxford Instruments Symmetry S2 detector (Oxford Instruments, Abingdon, UK), utilizing a 50 nm step size; data were processed with Oxford HKL’s Channel-5 software. The volume fraction of retained austenite was determined by X-ray diffraction (XRD) on a Rigaku DMAX-RB diffractometer (Rigaku Corporation, Tokyo, Japan) (Cu Kα radiation, λ = 0.15406 nm, 40 kV) following established procedures [31]. Room-temperature tensile tests were performed on dog-bone specimens (25 mm gauge length, 6 mm width) using an E45.305 MTS machine (MTS Systems Corporation, Eden Prairie, MN, USA) at a crosshead speed of 1 mm/min, with triplicate tests performed for reproducibility. Quasi in situ EBSD characterization was facilitated by interrupted tensile tests at various strain levels.

## 3. Results and Discussions

In Figure 1a, the initial microstructure of the steel is depicted, characterized by a typical lath martensitic microstructure with an average prior austenite grain size of 23 µm. The resulting microstructure after holding in the intercritical region at different heating rates, represented by Point 2 in Figure 1b, are shown in Figure 1d–f. The bright regions correspond to martensite that transformed from high-temperature austenite, while the dark regions represent ferrite. At a heating rate of 10 °C/s, a significant number of blocky structures are observed (Figure 1d), whereas a reduction in the heating rate to 1 °C/s results in fewer blocky structures and the formation of a fine film-like structure (Figure 1e). However, further reducing the heating rate to 0.1 °C/s causes the film-like structure to coarsen, with its width increasing from 0.2 to 1.0 µm (Figure 1e) and 0.6 to 2.0 µm (Figure 1f). The resulting microstructures after Q&P heat treatment of each investigated steel at Point 3 in Figure 1b are shown in Figure 1g–i. It was found that these microstructures inherit the morphological features during the intercritical region treatment at Point 2, though the boundaries are less distinct because martensite tempering and partial austenite decomposition occurred during partition [18,32]. The volume fraction of retained austenite (RA), as quantified from the XRD patterns in Figure 1c, exhibited a clear dependence on the heating rate. A maximum RA fraction of 19.0% was attained at the intermediate heating rate of 1 °C/s, in contrast to the lower values of 12.4% and 15.9% measured at 10 °C/s and 0.1 °C/s, respectively. This peak in RA retention at 1 °C/s correlates with the refined microstructure observed under this condition, which is posited to facilitate more efficient carbon enrichment of the austenite during the subsequent partitioning stage, thereby enhancing its stability against transformation.

**Figure 2:**
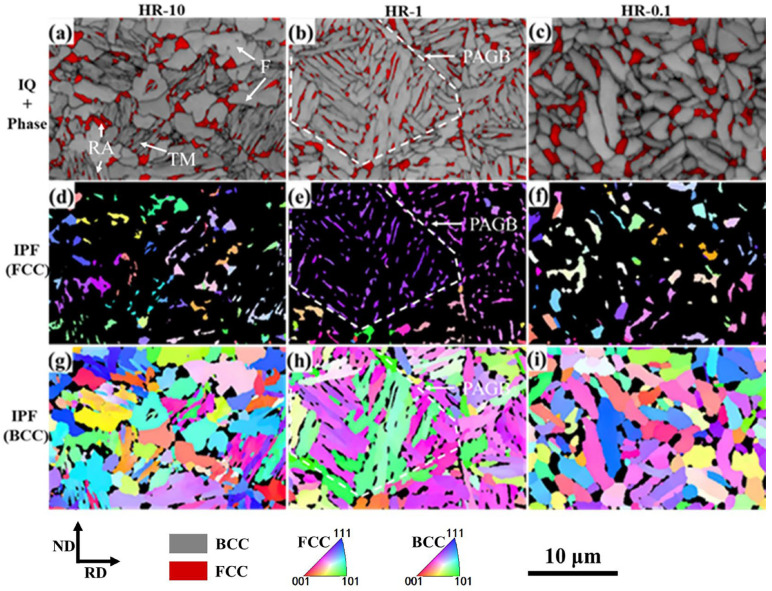
EBSD analysis of the microstructure at Point 3 in Figure 1b. (**a**–**c**) Image quality maps overlaid with phase color identification, where the BCC and FCC phases are shown in gray and red, respectively; (**d**–**f**) distribution maps of the FCC phase; and (**g**–**i**) inverse pole figure (IPF) maps of the BCC phase. RD and ND denote the rolling and normal directions, respectively.

Figure 2 shows EBSD images of HR-10, HR-1 and HR-0.1 steels. Both martensite and ferrite have a BCC (Body-Centered Cubic) structure, but martensite has more internal defects, which results in a lower IQ (Image Quality) value [7]. The EBSD analysis of the HR-10 specimen (Figure 2a,d,g) reveals a multi-phase microstructure comprising ferrite (F, light gray, high Image Quality), tempered martensite (TM, dark gray, low IQ), and retained austenite (RA, red). The RA exhibits dual morphology: blocky RA grains are predominantly situated at prior austenite grain boundaries or ferrite interfaces, while the film-like RA is consistently found interlacing the tempered martensite laths. This spatial distribution aligns with typical morphological characteristics reported for Q&P steels [13,33,34,35,36,37]. In comparison, in the HR-1 specimen (Figure 2b,e,h), the volume fraction of blocky RA is relatively low, with a significant proportion of the RA exhibiting a fine film-like morphology. Furthermore, in the HR-1 specimen, blocky TM is scarcely observed, with ferrite exhibiting an elongated shape, in contrast to the polygonal ferrite observed in HR-10. While the HR-0.1 specimen (Figure 2c,f,i) shares a general microstructural resemblance with HR-1, it exhibits conspicuous coarsening of both the retained austenite and ferrite phases. Furthermore, a key microstructural feature of the HR-1 specimen is the presence of clusters of retained austenite regions with nearly identical crystallographic orientations, as evident in Figure 2b,e,h. The spatial arrangement and orientation of these RA regions suggest that they nucleated and grew within a common prior austenite grain, the size of which is consistent with the initial microstructure shown in Figure 1a.

**Figure 3:**
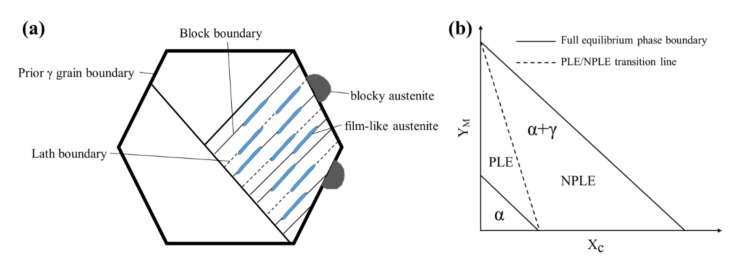
Schematic illustration of (**a**) nucleation sites for blocky and film-like austenite, and (**b**) isothermal sections of the Fe–C–M phase diagram in the two-phase region showing the PLE/NPLE boundary. Here, XC represents the mole fraction of carbon, and YM = XM/(1 − XC) denotes the site fraction of alloying elements.

The distinct RA morphologies in the HR samples originate from the kinetics of the reverse transformation from martensite to austenite. During intercritical annealing, austenite reversion from the lath martensite structure predominantly yields two morphologies: film-like and blocky, as schematized in Figure 3a [38,39]. Their formation is governed by competing growth models, dictated by temperature and composition (Figure 3b). Film-like austenite growth, which requires the partitioning of Mn and Si, is kinetically sluggish and adheres to the partition local equilibrium (PLE) model. In contrast, blocky austenite grows at higher temperatures in a near-partitionless manner, consistent with the negligible partition local equilibrium (NPLE) model. The transition between these modes occurs at a critical PLE/NPLE transition temperature (PNTT), calculated to be approximately 795 °C for this steel [40]. This mechanistic framework elucidates the observed microstructures. The rapid heating of HR-10 (10 °C/s) quickly surpasses the PNTT (795 °C), favoring the NPLE mode and thus the growth of blocky austenite at the expense of the film-like morphology (Figure 2a,d,g). Conversely, the slower 1 °C/s heating rate of HR-1 prolongs the duration below the PNTT, permitting the diffusion-controlled PLE growth of film-like austenite. The excessively slow rate of HR-0.1 (0.1 °C/s), while initially promoting film-like austenite, provides ample time for Ostwald ripening, leading to the coarsening seen in Figure 2c,f,i. Nucleation characteristics further differentiate these morphologies. Film-like austenite nucleates at martensite lath boundaries, maintaining a Kurdjumov–Sachs (K-S) orientation-based relationship with the surrounding matrix [41,42,43,44], which explains the clusters of similarly oriented RA in HR-1 (Figure 2b,e,h) that trace the prior austenite grains. Blocky austenite, however, exhibits multiple orientations within a single prior grain (Figure 2a,d,g), indicative of distinct nucleation sites. A detailed investigation of the RA orientation–property relationship, while promising, is reserved for future study.

**Figure 4:**
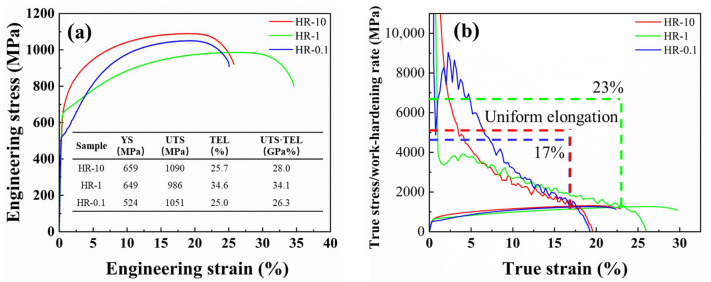
Mechanical performance of the investigated steels. (**a**) Engineering stress–strain curves and mechanical parameters (yield strength YS, ultimate tensile strength UTS, and total elongation TEL) of the HR-10, HR-1, and HR-0.1 samples and (**b**) corresponding true stress and work-hardening rate curves.

The tuned RA morphology greatly influences the mechanical performance of the samples. The engineering stress–strain curves of HR-10, HR-1 and HR-0.1 are shown in Figure 4a with their corresponding key mechanical properties. The HR-10 sample and HR-1 sample exhibit superior performance in yield strength, which is attributed to the elevated martensite content in the HR-10 sample and the ultrafine microstructure in the HR-1 sample. In comparison, the HR-0.1 sample possesses lower yield strength which in agreement with its coarsened microstructure. Meanwhile, the HR-1 sample demonstrates the highest uniform elongation, reaching nearly 23%, as shown in Figure 4b. The uniform elongation of metallic materials is ultimately limited by plastic instability (necking), the onset of which is universally described by the Considère criterion [45]. This criterion states that necking initiates when true stress surpasses the work-hardening rate. Therefore, exhibiting mildly sustainable work hardening (Figure 4b), the late onset of the plastic instability of HR-1 can be attributed to the elevated content of RA and the higher stability resulting from the film-like morphology of the RA. Moreover, at the late deformation stage (20–23%), the work-hardening rate of HR-1 still remains higher than 1000 MPa, which implies that even at high strains, a considerable amount of RA still experiences the TRIP effect. In comparison, the HR-10 and HR-0.1 samples show uniform elongations of only about 17%, as depicted in Figure 4b. Although both exhibit higher initial work-hardening rates than HR-1, their rates drop quickly after the early deformation stage (beyond 3% strain). This decline is mainly because HR-10’s blocky RA and the coarsened film-like RA in HR-0.1 lack sufficient stability. Consequently, TRIP effect peaks at the initial stage of deformation, boosting initial work hardening, but rapidly diminishes as strain increases. Therefore, as the strain increases, there is not enough stable RA to undergo the TRIP effect. Ultimately, the HR-1 sample achieves the highest product of tensile strength and elongation, reaching 34,116 MPa·%.

**Figure 5:**
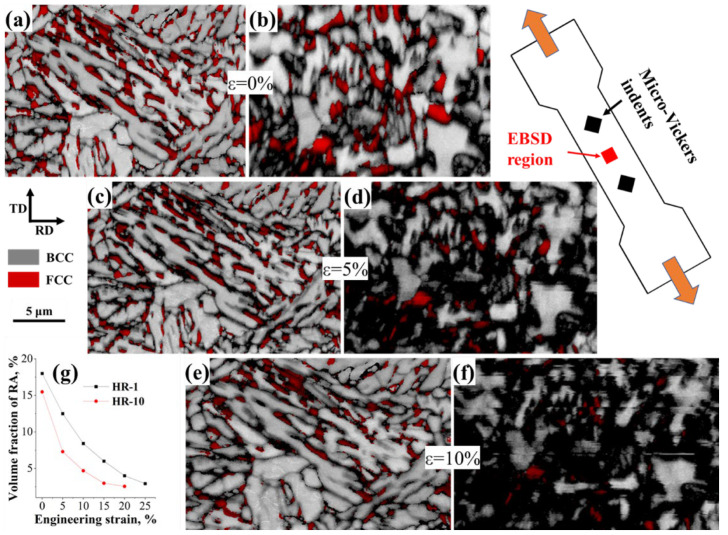
Quasi in situ EBSD observations showing the evolution of retained austenite (RA) stability in HR-1 and HR-10 specimens during tensile loading. IQ + phase maps of the ND sections at 0%, 5% and 10% strain are given in (**a**–**f**), while (**g**) summarizes the corresponding RA volume fraction derived from XRD measurements.

Quasi situ tensile characterization was conducted on HR-1 and HR-10 samples to investigate how the grain size and morphology of RA influence its phase stability and mechanical performance, as well as to trace its evolution during deformation. Figure 5a–f shows the deformation-induced martensite transformation in HR-1 and HR-10 specimens during tensile testing. Large-sized blocky RA transforms rapidly upon the onset of deformation, whereas the fine film-like RA remains stable until the later deformation stages. This behavior is attributed to the fine film-like RA’s higher interface energy, greater defect density, and its expected higher average carbon concentration due to more efficient partitioning from the surrounding martensite [46]. These factors, along with the fine grain strengthening effect, increase the macro-stress/strain threshold required to trigger its martensitic transformation [47]. During the early deformation stage, the HR-10 sample experienced a rapid transformation of its large blocky retained austenite into martensite, resulting in a higher initial work-hardening rate. In contrast, the HR-1 sample saw less transformation of its small film-like retained austenite, leading to a milder and earlier work-hardening rate (Figure 4b). However, due to its higher austenite stability, the HR-1 sample retained more austenite under large strain conditions. This allowed for continued transformation of retained austenite throughout deformation (Figure 5g), which boosted the work-hardening rate in later deformation stages and enhanced the material’s overall mechanical properties. These results demonstrate that the fine film-like retained austenite in the HR-1 sample possessed an optimal stability: it was sufficiently stable to avoid transforming prematurely at low strains, yet unstable enough to undergo a gradual transformation at high strains, thereby sustaining the TRIP effect over a broad deformation range and contributing to the superior mechanical properties.

## 4. Conclusions

In summary, by utilizing lath martensite as the initial microstructure and carefully regulating the annealing heating rate at 1 °C/s, blocky austenite formation was effectively suppressed, while coarsening of film-like austenite was controlled during two-phase region annealing in Q&P steel. Consequently, this approach yielded Q&P steel containing a high fraction (19.0%) of fine, film-like retained austenite, which demonstrated superior stability compared to blocky retained austenite. The fine film-like retained austenite contributed to a pronounced TRIP effect under significant deformation, leading to the excellent mechanical performance of 34.1 GPa%. Furthermore, while the optimized heating rate of 1 °C/s is readily achievable on many existing industrial continuous annealing lines, a detailed techno–economic analysis considering production throughput and the premium mechanical properties achieved would be a valuable subject for future industrial-scale research and development.

## Data Availability

The original contributions presented in this study are included in the article. Further inquiries can be directed to the corresponding authors.
